# 2632. Duration of viable SARS-CoV-2 shedding in immunocompromised COVID-19 patients during the widespread vaccination era

**DOI:** 10.1093/ofid/ofad500.2244

**Published:** 2023-11-27

**Authors:** Osamu Kanai, Kohei Fujita, Hiroaki Hata, Takao Odagaki, Tadashi Mio

**Affiliations:** National Hospital Organization Kyoto Medical Center, Kyoto, Kyoto, Japan; National Hospital Organization Kyoto Medical Center, Kyoto, Kyoto, Japan; National Hospital Organization Kyoto Medical Center, Kyoto, Kyoto, Japan; National Hospital Organization Kyoto Medical Center, Kyoto, Kyoto, Japan; National Hospital Organization Kyoto Medical Center, Kyoto, Kyoto, Japan

## Abstract

**Background:**

Individuals with COVID-19 who are immunocompromised have been reported to experience prolonged shedding of the severe acute respiratory syndrome coronavirus 2 (SARS-CoV-2) compared to those who are immunocompetent. However, it remains unclear how long viable SARS-CoV-2 shedding persists in immunocompromised individuals during the era of widespread vaccination against COVID-19. This study aimed to evaluate the association between the duration of viable SARS-CoV-2 detection among immunocompromised individuals and their COVID-19 vaccination status.

**Methods:**

We retrospectively reviewed patients who admitted to our institute for COVID-19 treatment between November 2022 and March 2023. We defined immunocompromised individuals as those who had received corticosteroids, immunosuppressants, or cytotoxic chemotherapy. We performed daily COVID-19 rapid antigen detection tests (RAD) on immunocompromised patients from 10 days after the onset of COVID-19 until two consecutive negative results were obtained.

**Results:**

Among 181 patients admitted for COVID-19 treatment, 22 immunocompromised patients participated in this study. Of these, 14 (64%), 6 (27%), and 10 (46%) patients received corticosteroids, immunosuppressants, and cytotoxic chemotherapy, respectively. Twelve (55%) patients were fully vaccinated. Nine (41%) patients required oxygen therapy for respiratory failure. All patients received antiviral therapy, with 5 (23%), 12 (55%), 5 (23%) patients receiving remdesivir, ritonavir-boosted nirmatrelvir, and molnupiravir, respectively. Three (14%) patients died from COVID-19. The median duration from COVID-19 onset to confirmation of negative RAD or death was 15.5 (interquartile range: 12 to 17) days. Until 20 days after the onset of COVID-19, 17 (77%) patients had achieved two consecutive negative RAD results. There was no association between vaccination status and the duration of positive RAD results (p = 0.59) or the proportion of patients achieving negative RAD results within 20 days of COVID-19 onset (P > 0.99).

**Conclusion:**

Most immunocompromised COVID-19 patients in our hospital achieved two consecutive negative RAD results within 20 days of COVID-19 onset. Vaccination status was not associated with the duration of detectable SARS-CoV-2 shedding.

**Disclosures:**

**All Authors**: No reported disclosures

